# Editorial: Nanomedicine for Deep-Tissue High-Resolution Bio-Imaging and Non-invasive Therapy

**DOI:** 10.3389/fbioe.2020.01002

**Published:** 2020-09-02

**Authors:** Yu Gao, Yanbo Pei, Ming-Yuan Wei

**Affiliations:** ^1^Key Laboratory for Organic Electronics and Information Displays and Jiangsu Key Laboratory for Biosensors, Institute of Advanced Materials, Jiangsu National Synergistic Innovation Center for Advanced Materials, Nanjing University of Posts and Telecommunications, Nanjing, China; ^2^Department of Physics, Harbin Institute of Technology, Harbin, China; ^3^Texas Commission on Environmental Quality, Austin, TX, United States

**Keywords:** nanomedicine, bio-imaging, drug delivery, ultrasound optical imaging, non-invasive therapy

The interdisciplinary research of nanotechnology and biomedical application yields an emerging field of nanomedicine, which seeks to develop functional agents for *in vivo* bioimaging (diagnosis), advanced drug delivery, and innovative therapy. It's our great pleasure to have this opportunity to include 16 articles in this Research Topic, either in-depth reviews or original research articles.

Nanomedicine for bioimaging is a contrast agent modified with functional moieties to target a specific part-of-interest, such as cancer cells or tumor tissues. Valuable *in situ* diagnostic information can be obtained only when both the bioimaging nanomedicine and the imaging instrumentation are communicating. Optical bioimaging techniques demonstrate advantages of low-cost, portability, and non-invasiveness. Yang et al. surveyed recent advances in the development of optically active nanomaterials, including gold, porous silicon, up-conversion, semiconductor nanocrystal, and carbon-based nanomaterials, along with mainstream optical bioimaging modalities, such as fluorescence, luminescence, surface enhanced Raman scattering (SERS), and photoacoustic (PA). Among them, fluorescence bioimaging holds great promise for the deep-tissue imaging but is hindered by the shallow imaging depth. Near infrared (NIR) fluorescent lifetime-based imaging can suppress the scattering and self-fluorescence noise and obtain the quantitatively functional information. Lian et al. overviewed the progresses of contrast agent's development (including organic dyes and nanomaterials) and the implementation of this imaging method. Cao et al. presented a detailed review on the fluorescent nanoparticles (NPs) working in near infrared wavelength ranging from 1,000 to 1,300 nm, which is called the NIR II window and in the range of which light is less absorbed by the tissue than in the visible and near infrared range below 1,000 nm. The authors summarized the characteristics of several typical nanomaterials including single-walled carbon nanotubes, Ag_2_S quantum dots, rare earth NPs, and organic fluorescent dye NPs. More recently, new bioimaging techniques that hybridize ultrasound and optics have attracted increasing attention, and deep-tissue high-resolution bioimaging has been conceived in a number of proof-of-concept studies (Pei and Wei, 2019).

The other idea to decrease the scattering and autofluorescence in the optical imaging is to use luminescent agents because the strong excitation light is eliminated. Li et al. summarized the progress on the development of NIR self-illuminating agents and the design principles. They also discussed the current challenges and future developments. Le et al. focused on the topic of chemiluminescence for optical imaging. The nanomaterials for bioluminescence and ultrasound enhanced chemiluminescence imaging are reviewed and the future direction is discussed.

Subsequently, a secondary nanomedicine that contains a therapeutic or regenerative drug will be deployed to the part-of-interest, and the drug release could be triggered precisely by non-invasive means, such as ultrasound, optics, magnet, or heat. Photothermal conversion agents triggers thermal therapy under the irradiation of the laser energy. Antibody (anti-protein A IgG) functionalized MoS_2_ nanosheets (MPPI NSs) developed by Zhang Y. et al. were demonstrated to be effective for the treatment of *S. aureus* focal infection on a mouse model. The MPPI NSs can specifically target to the bacterial and showed inactivation efficiency larger than 99.99% in both biofilms and in infected tissues, with minimal damage to mammalian cells. Meanwhile, the high extinction coefficient of MoS_2_ nanosheets in the NIR region provides the ability for real-time photoacoustic imaging. However, as mentioned previously, optical imaging and therapy usually suffer from low tissue penetration due to the absorption of the light by the tissue. For deep-seated tumor such as hepatocellular carcinoma, Zhou et al. labeled radionuclides ^125^I to the AA98 monoclonal antibody (^125^I-AA98 mAb) against CD146, a biomarker for angiogenesis with high expression in hepatocellular carcinoma cells. After intratumoral injection in a mouse model, the ^125^I-AA98 mAb not only inhibited the early angiogenic process, but also induced apoptosis of the cancer cells, achieving higher therapeutic efficacy than treatment by free ^125^I. The tumor response to the targeted therapy were monitored by single-photon emission computerized tomography (SPECT) with high sensitivity and tissue penetration. To overcome the limitation of penetration depth of light, ultrasound can be used for both imaging and therapy. In the research by Vighetto et al., the generation of reactive oxygen species (ROS) by single-crystalline zinc oxide (ZnO) NPs and ultrasound were studied. The cavitation effect of gas nanobubbles under ultrasound were responsible for the generation of hydroxyl and superoxide anions, which causing ROS-mediated apoptosis or necrosis of cells. Notably, the generated acoustic signal can be monitored by B-mode ultrasound imaging, suggesting the capability of ZnO NPs as an ultrasound aided theranostic agent. Interestingly, the health effects of non-invasive therapy on human should be carefully studied. Song et al. demonstrated that whole body vibration (WBV), a non-invasive physical therapy, significantly increased the CD4 and CD25 positive lymphocytes as well as the population of Treg cells in the spleen. Therefore, the contents of a variety of bacteria changed, such as *Lactobacillus animalis* in mice, and *Lactobacillus paraplantarum* and *Lactobacillus sanfranciscensis* in human.

In a best-case scenario, the therapy or regeneration process can be monitored real-time through the bioimaging. The interactive cooperation between these two procedures will provide accurate and effective treatment for human diseases. Kuriakose et al. developed a new biodegradable photoluminescent polylactones-co-poly (lactic-co-glycolic acid) copolymers (BPLP-PLGA) as nanocarriers for protein delivery. These NPs displayed superior stability (2 days) in physiological conditions, tunable release kinetics and fluorescence emission, compatibility with endothelial cells, and good hemocompatibility. Thereby, the BPLP-PLGA NPs have potential to serve as optical contrast agents and nanocarriers of the drugs for cardiovascular diseases. Tian et al.'s mini-review emphasizes some recent advances of gold nanomaterials-based photothermal imaging (PTI), SERS imaging, and PA imaging-guided *in vivo* therapy in NIR region (>800 nm). Hu et al. examined the potential value of multimodality gadolinium-based NPs (AGuIX) for non-invasive theranostic MRI-guided radiotherapy in hepatocellular carcinoma (HCC). The AGuIX provide better detection of tumors in imaging, and precise identification for accurate MRI-guided radiotherapy; meanwhile, the heavy elements in this novel nanoparticle can enhance radiosensitizing effect by irradiation dose deposition.

We are extremely excited to notice that artificial intelligent (AI) has been pushing the development of nanomedicines. For instance, with the rapid development of nanotechnology in the field of biomedicine, artificial blood, or blood substitute has shown promising features for the emergency treatment of BDDs. Chemotherapy, bone marrow transplantation, and stem cell therapy have been used to treat blood disorder diseases (BDDs). However, the cure rates are still low due to the availability of the right type of bone marrow and the likelihood of recurrence and infection. Zhang N. et al. surveyed recent advances in the development of artificial blood components: gas carrier components (erythrocyte substitutes), immune response components (white blood cell substitutes), and hemostasis-responsive components (platelet substitutes). Platelet-inspired nanomedicines for cancer treatment were also discussed.

Wang et al. summarized the applications of nanozymes for disease imaging and detection to explore their potential application in disease diagnosis and precision medicine. Compared to natural enzymes, nanozymes exhibit the unique advantages including high catalytic activity, low cost, high stability, easy mass production, and tunable activity. In addition, as a new type of artificial enzymes, nanozymes not only have the enzyme-like catalytic activity, but also exhibit the unique physicochemical properties of nanomaterials, such as photothermal properties, superparamagnetism, and fluorescence, *etc*. Despite the remarkable advantages of nanozymes, there remains plenty of limitations while put nanozymes into practical clinical application, such as poor dispersibility, easy sedimentation after surface modification, limited catalytic types, poor substrate selectivity, and potential nanotoxicity.

Compared to detecting proteins secreted by tumors, detecting secreted miRNAs has become more attractive for monitoring tumor progression. Ma et al. designed an artificial intelligent signal amplification (AISA) system including double-stranded SQ (S, signal strand; Q, quencher strand) and FP (F, fuel strand; P, protect strand) according to thermodynamics principle for sensitive detection of miRNA *in vitro* and *in vivo*. The design features conceiving signal amplification and preventing from *in vivo* degradation and complications. Based on this detection system, the precancerous lesions of liver cancer were diagnosed and reconstructed.

The last but not the least, we should always keep in mind to take into consideration of the potential nanotoxicity of nanomedicines, which may fail the clinical trial due to severe side effects. To this end, Dong et al. investigated the candidate fate of acid-oxidized single-walled carbon nanotubes (SWNCTs) in non-activated primary mouse peritoneal macrophages (PMQ). All data showed that exocytosis, uptake, biodegradation, and sustainable retention of SWCNTs co-exist in primary macrophages.

In summary, we are pleased to witness such landmark progresses in the development of nanomedicines that are aiming to provide real-time bioimaging-guided non-invasive therapy for human diseases, along with the integration of artificial intelligence technology. The findings included in this Research Topic will open the avenue for brighter ideas in future “Smart Nanomedicines” development. During this pandemic season of 2020, we shall keep hope and trust in science, only by which we can overcome the challenge from the COVID-19.

## Author Contributions

YG, YP, and M-YW wrote and edited the manuscript. All authors contributed to the article and approved the submitted version.

## Conflict of Interest

The authors declare that the research was conducted in the absence of any commercial or financial relationships that could be construed as a potential conflict of interest.
